# Orienting attention across binocular disparity

**DOI:** 10.1093/pnasnexus/pgad314

**Published:** 2023-09-27

**Authors:** Baptiste Caziot, Martin Rolfs, Benjamin T Backus

**Affiliations:** Graduate Center for Vision Research, SUNY College of Optometry, New York, NY 10036, USA; Neurophysics Group, Philipps-Universität Marburg, Marburg 35043, Germany; Center for Mind, Brain and Behavior (CMBB), Philipps-Universität Marburg and Justus–Liebig–Universität Gießen, Marburg 35032, Germany; Bernstein Center for Computational Neuroscience and Department of Psychology, Humboldt University Berlin, Berlin 10099, Germany; Graduate Center for Vision Research, SUNY College of Optometry, New York, NY 10036, USA; Vivid Vision, Inc., San Francisco, CA 94103, USA

## Abstract

The spatial distribution of covert visual attention following an exogenous cue is often described as a spotlight, which disregards depth. Here, we study the orienting of attention across binocular disparity, a key depth cue in primates. A small Gabor patch target was displayed at ±12-arcmin horizontal offset in each eye independently, resulting in four possible 3D locations. With some latency relative to target onset (0–300 ms), an attentional cue was displayed at one of five binocular locations, resulting in various combinations of relative azimuth (horizontal position) and disparity (depth). Observers’ task was to discriminate the orientation of the target. Observers’ performance decreased as the relative azimuth between the cue and the target increased. Performance also decreased with the difference in disparity, even when the azimuth remained constant. Performance varied with the delay between the cue and the target and was maximal between 100 and 150 ms. The orienting of attention in azimuth and depth followed the same time course. We mapped the 3D shape of attentional focus over time and found that the spatial envelope was approximately a Gaussian modulated in time. These results could not be explained by monocular confounds nor by eye movements. We conclude that exogenous cues direct attention not only to their visual direction but also to their depth and that binocular disparity is sufficient to define that depth. The identical time course and interaction between azimuth and depth suggest a shared mechanism, and therefore that visual attention to spatial location is an intrinsically 3D process.

**Significance Statement**

Visual attention is often described as a spotlight, which disregards depth of information within the visual scene. Here, we show that attention can be oriented across binocular disparities, a depth cue. We mapped the shape of the attentional focus across both azimuth and depth. We find an interaction between azimuth and depth, suggesting that attended locations are best described as a 3D ovoid shape. The time course of the deployment of attentional selection is similar across dimensions. We further show that these results cannot be explained by vergence eye movements. We conclude that covert visual attention is intrinsically a 3D process.

## Introduction

Visual attention is routinely captured by salient events in the environment (a door that opens, a reflection seen in the distance, and a person turning toward you). Studies of attention using such “exogenous” cues (usually a brief flash) show consistently that discrimination ability at the cued location improves compared to uncued locations [1]. The spatial distribution of this attention is often described as a spotlight [1–3], which disregards depth of information within the visual scene. Extremely little is known about the orienting of attention in depth, and current models of attention [4–7] do not take the 3D layout of visual scenes into consideration.

The few prior studies that investigated attention in depth used the two main paradigms in the field of attention: visual search [8] and cueing [2]. For visual search, Nakayama and Silverman [9] found that a target that differs from distractors in disparity pops out in a visual search task, which is usually interpreted as an orienting of attention [10, 11]. In this situation, disparity can be thought of as a stimulus feature that attracts the attentional spotlight by contributing to a 2D saliency map, like other features that attract attention such as color, orientation, etc. [7]; it remains unknown whether the target's discrepancy in depth caused cueing to its particular depth plane. The second approach, cueing in depth, did show that attention can be oriented to a given depth layer based on monocular [12] or binocular cues [13], creating a 2D spotlight in a specific depth plane. However, the temporal and spatial distribution of the cued attention and particularly the relationship between depth and visual direction have not been examined.

**Fig. 1.**
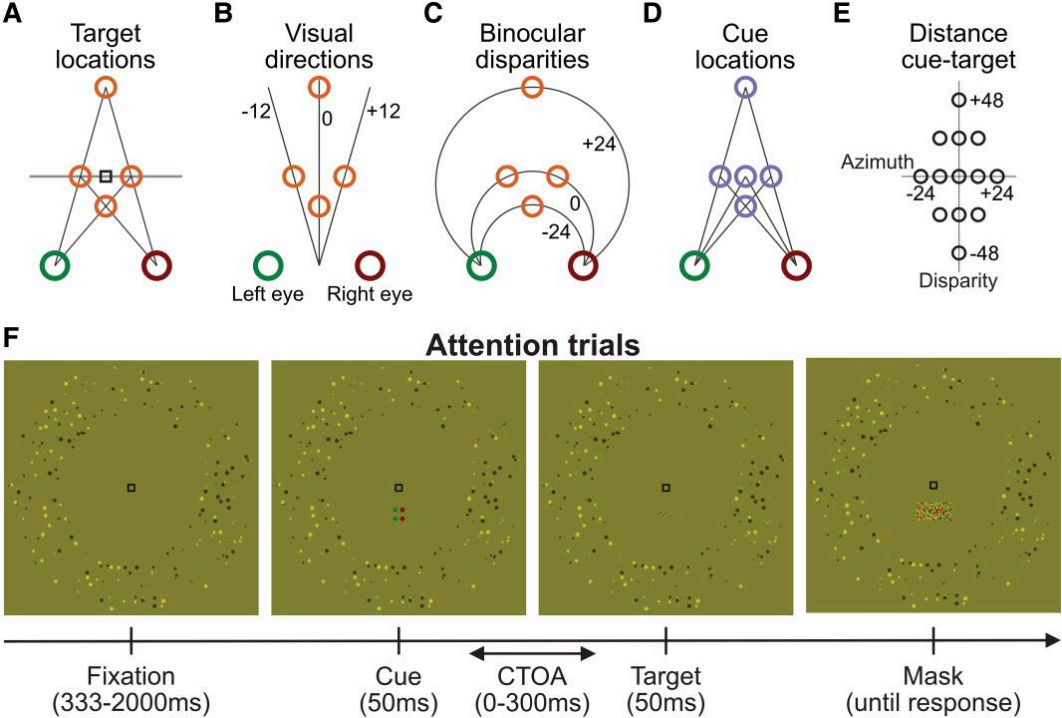
A) Gabor patch targets were displayed 12 arcmin to the right or left of fixation (black square) independently for each eye, resulting in four possible binocular locations (orange circles): left, right, near, or far. B) The four target locations had three visual directions (−12, 0, or +12 arcmin, black lines) and (C) three binocular disparities (−24, 0, or +24 arcmin; black arcs represent Vieth–Müller circles). D) Cues were displayed at five possible binocular locations (purple circles): same as the targets or directly above or below fixation. E) This geometry produces 13 possible distances between the cue and the target (black circles) including the same location (origin), pure change in visual direction (*x*-axis), pure change in disparity (*y*-axis), or mixed change in visual direction and disparity (off-axes). F) Time course of a trial. Here, for demonstration purposes, stimuli are depicted by anaglyph displays rather than by Wheatstone stereoscope stimuli (as were used in the experiments). Red points are visible by the right eye through a green filter, but green points are invisible. The opposite is true for the left eye looking at the stimulus through a red filter. After a random fixation period, the cue (two dots flanking one of the locations) was displayed for 50 ms, and after a delay (CTOA) of 0 to 300 ms, the target (an oriented Gabor patch) was displayed for 50 ms. In this example, the cue is valid (at the same location as the target). Targets were immediately masked by white noise uncorrelated between the two eyes, and observers reported the orientation of the target (clockwise vs counterclockwise). Throughout the trial, observers were required to fixate the black fixation mark at the center, and high-contrast fusional aids were displayed around the stimulus to help maintain vergence eye posture. Additional stimulus configuration examples are shown in Fig. S1.

In this study, we displayed a cue prior to a target, at locations that differed in both azimuth (horizontal angular distance) and disparity (depth; see Fig. 1). The various differences in azimuth and disparity allowed us map an attentional field extending in depth. We also varied the Cue-to-Target Onset Asynchrony (CTOA) (i.e. the delay between the cue and the target), because attention rises and falls over time after an exogenous cue is presented [1]. Our results show an interaction between the orienting of attention in azimuth and disparity, which had a similar time course. Overall, these results suggest that attention is an intrinsically 3D phenomenon and that the disparity of a cue is sufficient to mediate the orienting of attention in depth.

## Results

Observers were instructed to discriminate the orientation (clockwise or counterclockwise) of a small Gabor patch target subtending approximately 30 arcmin. The target was displayed 1.5° above or below fixation and with ±12 arcmin to the right or left in each eye independently, resulting in four possible binocular locations (neglecting whether it was above or below fixation): right, left, close, and far. With a delay varying between 0 and 300 ms prior target onset (CTOA), a cue consisting of two high-contrast black dots flanking the target was displayed at one of five possible binocular locations uncorrelated with target location: the same four locations as the targets or a fifth, central location. Both the cue and the target were displayed for 50 ms. Immediately after target offset, a high-contrast white-noise mask, uncorrelated between the two eyes (therefore eliciting no specific disparity), was displayed over a large area containing all possible target and cue locations until response.

### Orienting of attention in depth

**Fig. 2.**
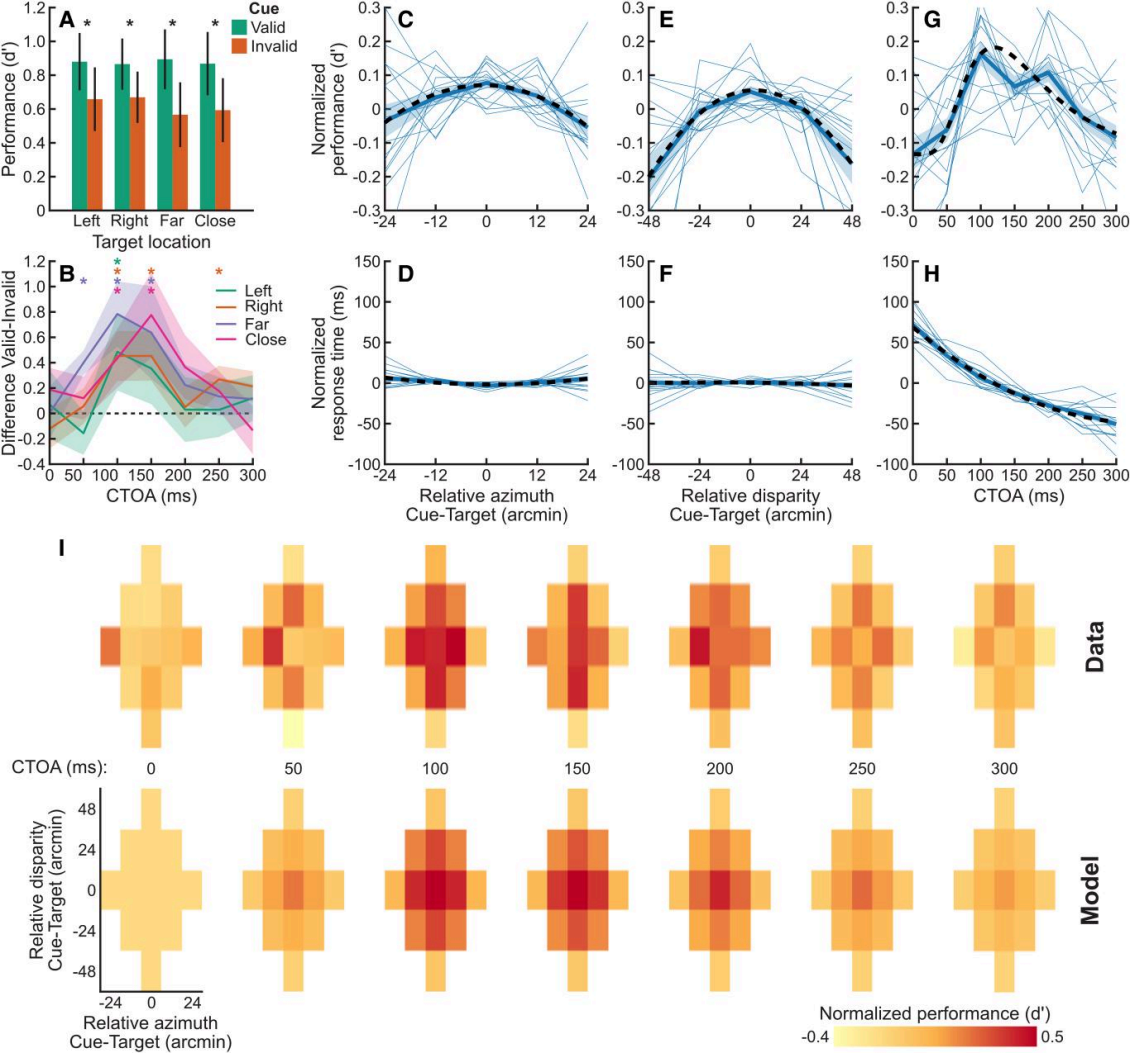
A) Performance (*d*′) as a function of target location (abscissa) and cue validity (color). Asterisks indicate significant differences between valid and invalid conditions (see text for *P*-values). B) Mean difference between valid and invalid conditions (*d*′) across target locations (colors) and standard errors (shaded areas). Stars indicate differences significantly larger than 0. C) Normalized performance (*d*′) as a function of relative azimuth between the cue and the target, marginalized over disparity and CTOA. Thin lines are individual observers; thick blue line and shaded area are mean and standard error of the mean across observers, respectively. The dashed line is a polynomial fit. D) Same as C for normalized response times (ms). E) Same as C for performance as a function of relative disparity, marginalized over azimuth and CTOA. F) Same as E for normalized response times. G) Same as C for performance as a function of CTOA, marginalized over azimuth and disparity. The dashed line is a log-normal fit. H) Same as G for normalized response times. I) Normalized performance (color scale) plotted as function of relative azimuth (abscissa) and disparity (ordinates) between the cue and the target and as a function of the CTOA (lattice plots). The top row is data averaged across observers, and the bottom row is the prediction from the best probit model.

On “valid” trials, the cue was displayed at the same location as the target in both eyes (e.g. cue-right/target-right or cue-far/target-far); on “invalid” trials, the cue was displayed at the other location in both eyes (e.g. cue-left/target-right or cue-close/target-far). Figure 2A plots performance (visual sensitivity, *d′*) as function of target location and cue validity. Performance was significantly higher when the cue was valid than invalid at all four target locations (difference of resampled performance; *P* = 0.043, *P* = 0.001, *P* = 0.001, and *P* = 0.002 for target locations left, right, far, and close, respectively). To get an estimate of the time course of this effect, we then analyzed the difference in performance between the valid and invalid conditions as a function of the CTOA, which varied between 0 and 300 ms. Figure 2B plots these differences as a function of CTOA for the four target locations. Performance differences start near 0 for a CTOA of 0 ms, rise to approximately 0.4 and 0.8 *d′* differences for CTOAs of 100 and 150 ms, and then return to near 0 for CTOAs of 250 and 300 ms. The difference between valid and invalid conditions was significantly higher than 0 at all target locations for a CTOA of 100 ms, at all but the left target location at 150 ms (resampling of the differences, *P* < 0.05, uncorrected for multiple comparisons). This difference was also significant for the far target location at 50 ms and for the right target location at 250 ms, the latter being most likely a spurious statistical difference. To compare the time course of the orienting of attention in depth as compared to azimuth, we resampled the data set and fitted a log-normal function to the difference between valid and invalid conditions for each sample. The distributions of modes and standard deviations of the log-normal fits were not significantly different between any of pairs of target locations (*P* > 0.05).

For a more fine-grained analysis, we looked at all possible differences in azimuth and disparities (see Fig. 1E). Figure 2C shows performance, normalized by the observer's mean performance, as a function of the difference in azimuth (horizontal position) between the cue and the target. Performance peaks at a difference of 0 and decreases as the difference of azimuth increases, indicating that performance is maximal when the cue was displayed at the same azimuth as the target. To assess significance, we resampled the curves across observers and fitted a second-order polynomial to each sample (see Fig. S2). While the peak value of the polynomial fit occurred at an azimuth that was not significantly different from 0 (*P* = 0.58), the quadratic term was significantly lower than 0, indicating that performance decreased symmetrically with the difference in azimuth (*P* = 0.02). This effect in performance was mirrored by a small effect in response times (Fig. 2D), for which the same polynomial fit had a second-order term significantly higher than 0 (*P* = 0.03). Thus, the decrease in performance is associated with a slight increase in response times, and it cannot be explained by a speed–accuracy trade-off.

Similarly, normalized performance as a function of relative disparity between the cue and the target tends to decrease with increasing differences in position between the cue and the target (Fig. 2E). Again, a significantly negative second-order term corroborated this decrease in performance (*P* < 0.001; see Fig. S2). The peak location occurred at a disparity that was not significantly different than 0 in a two-sided test (*P* = 0.076), although the data do not rule out an asymmetry between crossed and uncrossed disparities for the orienting of attention, with larger performance benefits for uncrossed disparities. Response times (Fig. 2F) were not modulated by the relative disparity between the cue and the target, and the second-order term of the polynomial fits was not significantly different from 0 (*P* = 0.5).

Finally, Fig. 2G shows normalized performance as a function of CTOA. Performance increased with delay, peaking between 100 and 150 ms, then decreasing with further delay and reaching baseline by 300 ms. To assess significance, we fitted a log-normal function to resampled data and found that the amplitude of the log-normal function was significantly larger than 0 (*P* < 0.001). This function reaches its peak at 118 ms, indicating that performance peaks between 100 and 150 ms. Response times were strongly modulated by the CTOA (see Fig. 2H), dropping over 100 ms between the CTOA of 0 ms (cue and target appear at the same time) and 300 ms. We fitted polynomial functions to response times (RTs) and found that the first- and second-order terms are significantly lower than 0 (*P* < 0.001) and the peak value was significantly higher than 300 ms (*P* < 0.001) indicating that RTs decreased continuously with CTOA.

### Interactions between azimuth and depth

Finally, we looked at the full interaction between the difference in azimuth, disparity, and onset between the cue and the target. Figure 2I plots performance (color-coded) as a function of relative azimuth (abscissa) and disparity (ordinates) between the cue and the target plotted in the same coordinate system as Fig. 1E. Here, data are decomposed in 91 conditions and are therefore noisy. However, as suggested by prior analyses, performance is clearly modulated by the CTOA, with maximum performance for CTOAs of 100 and 150 ms. Similarly, performance is maximal near the center of the field, where the cue azimuth and depth match that of the target.

To analyze these effects, we resampled the data and fitted a probit model to each sample. We compared various probit models and used cross-validation for model selection (see Materials and methods and Figs. S3 and S4). The model with the highest cross-validation score assumed that performance is a multivariate Gaussian modulated by the CTOA. This model had a better cross-validation score than a model that assumed that the effects of disparity and azimuth on performance are independent, suggesting that the focus of attention is better described as a 3D ovoid rather than the intersection of two foci for spatial locations and depth. Similarly, this model had a higher cross-validation score than a model assuming a different time course for the orienting of attention in azimuth and depth.

### Eye movements

**Fig. 3.**
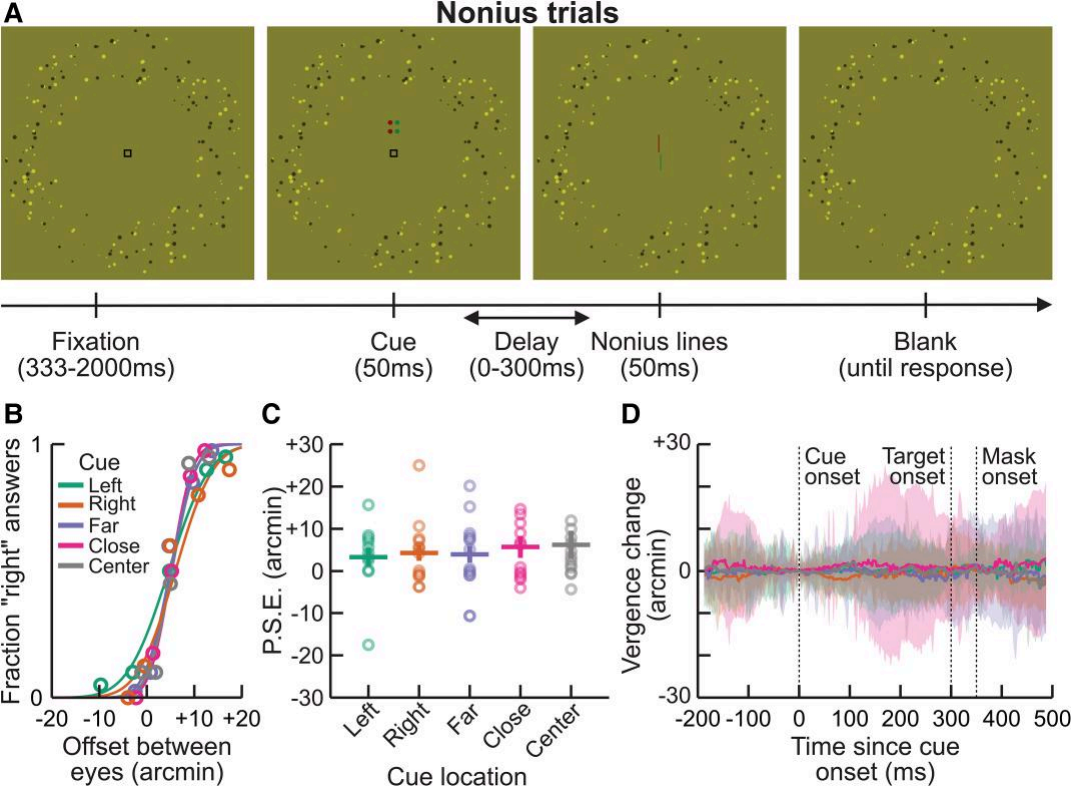
A) Time course of a Nonius task trial. After a random fixation period, the cue was displayed for 50 ms similar to regular trials. After a fixed delay of 150 ms, monocular vertical lines with a horizontal offset were displayed for 50 ms and then blanked until response. As in Fig. 1F, the right eye sees red points through a green filter but not green points and conversely the left eye through a red filter. Observers had to report whether the top line was to the right or left of the bottom line. B) Example of psychometric functions as a function of the offset between the monocular lines (abscissa) and cue location (color) for one observer. C) Individual (circles), mean, and standard error (lines) of the psychometric functions PSEs as a function of cue location. D) Average change in vergence eye posture during attentional trials across observers (lines) relative to cue onset and standard error (shaded area) as a function of time (abscissa) and cue location (colors). Only data for the longest CTOA (300 ms) are plotted for clarity; see Fig. S5 for other CTOAs.

To be certain that the effects of cueing were mediated by covert attention rather than by changes in vergence eye posture, we monitored vergence eye posture and compared it across the different cueing conditions. Throughout the experiment, we monitored eye movements both behaviorally and with eye tracking. In a subset of “Nonius trials,” randomly interleaved with attentional trials, the fixation mark disappeared 150 ms after cue onset (when the effect of attention was expected to be strongest) and was replaced by vertical monocular lines with a horizontal offset (see Fig. 3A). To the observers, these stimuli appeared as a Vernier acuity task and observers had to report whether the top line was to the right or left of the bottom line. The horizontal offset was controlled by an adaptive procedure. We fitted psychometric functions (see Fig. 3B) to estimate the point of subjective equality (PSE) at which the monocular lines appeared aligned vertically, and thus match the vergence eye posture of the observers. Figure 3C shows the mean PSE as a function of cue location. These PSEs are significantly higher than 0 at each cue location (*t*-tests, *P* < 0.01) but are not different from each other in a one-way ANOVA (*F*(14) = 0.34, *P* = 0.85). The difference between the PSEs in the near and far cued location was also not significantly different from 0 in a pairwise *t*-test (*t*(14) = 0.71, *P* = 0.49).

Figure 3D plots change in vergence angle measured through oculometry, averaged across observers, during the attentional trials with a CTOA of 300 ms as a function of time relative to cue onset. Vergence at cue onset was subtracted for each trial to test specifically whether the cue location changed vergence eye posture. The mean vergence eye posture during stimulus presentation did not significantly deviate from 0 (starting position) for any CTOA (resampling of the mean, *P* > 0.05, uncorrected for multiple comparisons). The difference in vergence eye posture between the near and far cue positions was also not significantly different from 0 for any CTOA (*P* > 0.05).

## Discussion

Here, we investigated the orienting of attention in depth as defined by binocular disparities. While this topic has attracted interest from the field, results have been contradictory. For instance, multiple studies [14–21] either failed to replicate or strongly limited the generality of the classical result from Nakayama and Silverman [9]. Similarly, while some studies have reported an effect of depth cueing in cued attention experiments [22–27], others did not [28–31]. Most importantly, prior studies have focused on whether attention can be oriented toward a given depth layer rather than treating the visual environment as a proper 3D space.

To address these inconsistencies in the literature, we developed a robust paradigm and advanced the study of attention in depth in several important ways. First, we used a carefully controlled technique largely devoid of monocular confounds. Second, we tested multiple relative azimuths and depths, allowing us to measure interactions between azimuth and depth. Third, we measured the time course of attention using multiple CTOAs. And finally, we carefully measured eye position, both behaviorally (Nonius lines) and through oculometry, to eliminate possible eye movement confounds.

Overall, our results show a clear orienting of attention in depth. As the position of the cue was uninformative about the position of the target, we can conclude that this effect is automatic. The different relative azimuths and depths of the cue relative to the target allowed us to measure possible interactions between these components. We found that observers’ performance was better explained by a model where attention is oriented to the 3D location of the cue rather than an independent orienting of attention to a 2D location of the cue and its depth (see Figs. S3 and S4). This and the identical time course of the orienting of attention in azimuth and depth strongly suggest that attention is intrinsically a 3D phenomenon. This is in contrast with models that posit a role for disparity as a simple cue to a location on a 2D saliency map [7] or to surfaces rather than 3D locations [9, 32].

At a viewing distance of 114 cm, the disparity of 24 arcmin in our stimuli corresponded geometrically to a depth of approximately 15 cm. In contrast, an azimuth of 12 arcmin corresponded to a lateral distance of only 0.4 cm, confirming that attention can be very narrowly tuned to spatial locations [33]. Here, the 3D focus of attention was far more narrowly tuned for azimuth than depth; however, disparity maps nonlinearly with distance at different viewing distances [34]. Mapping the spatial extents of the attentional focus at different viewing distances would allow dissociating whether attention acts at a 3D perceptual level or prior to disparities being converted to distance.

Binocular vision is commonly believed to be slow [35, 36], which would limit its use to conscious appreciation of depth or slow tasks such using precision tools [37]. We have shown that binocular disparities are in fact processed as quickly as luminance signals [38–40] and have argued that they could contribute to early visual processes, such as the orienting of attention, more than has been previously appreciated. The identical time course of the orienting of attention in azimuth and depth is in good agreement with this hypothesis. That attention seems to be an intrinsically 3D process, and that disparity is sufficient to define that depth opens intriguing and potentially fruitful lines of research. Many known deviations exist between stereoscopic and monocular “2D” vision, such as different spatiotemporal limits [41] or dissociate triggering of conjunctive and disjunctive eye movements [42, 43], which could lead to a better understanding of attention in general.

**Fig. 4.**
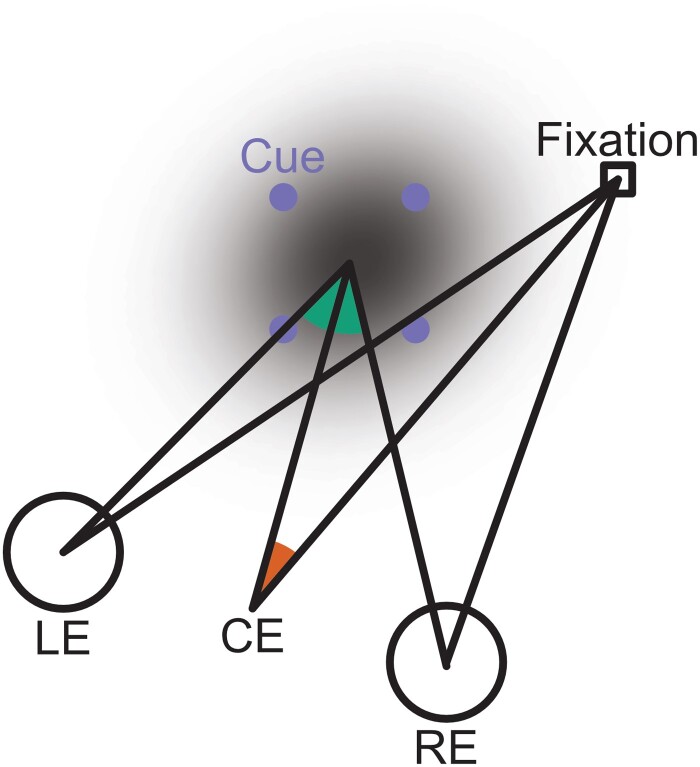
Cartoon depiction of hypothetical ovoid-shaped attentional benefit in 3D space after a 3D location is cued. LE, RE, and CE are respectively left eye, right eye, and cyclopean eye. Here, an exogenous attentional cue (purple dots) attracts the 3D focus of attention to its location. Attentional benefits are limited not only to the azimuth (and/or elevation) of the cue (orange angle) but also to the cue disparity (green angle).

Overall, our results suggest that describing attention as a spotlight, while a useful metaphor in a research context, is inappropriate in the context of 3D scenes. We suggest that attention takes an ovoid shape in 3D (see Fig. 4). In this study, both the stimulus array and attentional ovoid had small spatial extents, on the order of arcmin. However, this ovoid might inflate in response to task demands in natural environments, such as cueing at a more eccentric visual direction or greater disparity from fixation by an object that starts to move within the visual field.

## Materials and methods

### Observers

Observers were 15 students and faculty at the SUNY College of Optometry, including the first author. All observers had normal or corrected-to-normal vision and a stereoacuity of 20 arcsec or better as measured with the Randot stereoacuity test (Precision Vision, La Salle, IL, USA). All but the author were naïve to the purpose of the experiment. The study was conducted in agreement with the Declaration of Helsinki and approved by the Internal Review Board of SUNY College of Optometry (250859—cue reliability and depth calibration during space perception).

### Apparatus

The experiment was carried on a Wheatstone (mirror) stereoscope mounted on a metal beam. Two small first-surface mirrors on a two-axis tilt control mount (Edmund Optics, Barrington, NJ, USA) were placed in front of each eye at a 45° angle. Two larger first-surface mirrors were placed more laterally on each side, also at 45° and aligned with the center of the corresponding half-monitor. For demonstration purposes, in Figs. 1 and 3, stimuli are instead generated for anaglyphs.

Stimuli were displayed on a monitor VG248QE (ASUS, Taipei, Taiwan) at 114.6 cm from the observer. Stimuli were displayed for the right eye in the right half of the monitor and in the left half for the left eye, resulting in an effective resolution of 960 × 1,080 pixels at 120 Hz. A black opaque horizontal divider was placed between the two eye mirrors to prevent each eye from seeing the other half of the monitor. At the beginning of the experiment, this divider was removed and vertical and horizontal lines were displayed at the center of the monitor and at the center of one half of the monitor for each eye independently. Observers were instructed to use the screws on the eye-mirror mounts to adjust the position of the mirror such that the two crosses formed by the lines appear fused. This was done to ensure that the observer's vergence eye posture, when looking at the fixation through the stereoscope, matched the vergence eye posture they had when looking at the center of the monitor directly. The distance in optical pathlength between direct viewing and viewing through the mirrors caused the accommodative distance to be 12 cm more than the vergence distance, but no observer reported any visual discomfort during the experiment.

An Eyelink 1000+ eye tracker (SR-Research, Ottawa, Canada) was positioned approximately 45 cm away from the observers. It was placed in a way that allowed it to capture the image of the eyes from below the stereoscope mirrors. Eye position was monitored in both eyes at 1,000 Hz. At the beginning of the experiment, the eye tracker was calibrated with a nine-dot calibration matrix for each eye independently.

### Stimulus

Observers were asked to discriminate the orientation of a small target Gabor patch (Gaussian envelope *σ* = 6 arcmin, carrier frequency = 5 cycle.deg^−1^) oriented ±45°. Pixel noise with normally distributed contrast was added to the carrier to vary the signal-to-noise ratio (SNR). The overall contrast of the Gabor patch was kept constant; thus, the amplitude of the signal and noise was (with SNR in dB):


$${\mathrm{RMS}}_{\mathrm{noise}}=\sqrt{\frac{E}{1+10^{\frac{\mathrm{SNR}}{40}}}}\;\mathrm{and}\;{\mathrm{RMS}}_{\mathrm{signal}}=\sqrt{E-{{\mathrm{RMS}}_{\mathrm{noise}}}^{2}}.$$

*E* was set to a fixed contrast. Example stimuli at different SNRs are shown in Fig. S6. The target was presented at a position that was either 1.5° above or below the fixation point and in each eye independently 12 arcmin to the right or to the left of the fixation mark, resulting in four possible binocular locations (neglecting whether it was displayed above or below fixation): right, left, closer, or farther (see Fig. 1).

Prior to the target, a cue, two small black dots (4 arcmin), was displayed 15 arcmin above and below the elevation of the target. The binocular location of the cue was one in five: the same four binocular locations as the target or a fifth central location vertically aligned with fixation. The cue was *always* displayed in the same general direction as the target (below or above fixation), but the relative azimuth and disparity of the target relative to the cue varied in 20 combinations from trial to trial (4 target locations × 5 cue locations).

Finally, the CTOA was varied randomly between 0 and 300 ms (uniform distribution, 50-ms steps). Overall, there were 140 different combinations of relative binocular locations and CTOA between the cue and the target for which we collected 15 trials per session (2,100 trials total). Most observers collected two sessions, but three observers collected only one.

In alternation with attentional trials, Nonius trials were performed to monitor the observers’ vergence eye posture. In these trials, the cue was displayed to one of the five possible cue locations, but instead of displaying a Gabor target, the fixation disappeared and was replaced by two monocular vertical lines 60 × 3 arcmin, one above and one below fixation. To the observers, the Nonius lines appeared as a Vernier acuity task, and they had to report whether the top line was to the right or left of the bottom line. The gap between these monocular lines was controlled by a Psi adaptive method [44].

### Procedure

The Gabor target's SNR was staircased for each individual. At the beginning of the experiment, we estimated the observer's threshold of 90% in 200 trials using the Psi adaptive method [44]. During threshold estimation, the CTOA was 150 ms, presumably when performance would be maximal, and the target was displayed at the central location, a location that was not used during the actual experiment. The median SNR across individuals was −26 dB.

Once the threshold was estimated, the observer performed 20 blocks of approximately 130 trials. The exact number of trials per block varied as the Nonius trials were randomly interleaved with attentional trials. One recording session lasted about 1.5 h.

### Analysis

Performance was estimated by converting the fraction of correct answers per condition and participant into *d′* [45]. Fits were performed using the Nelder–Mead algorithm to find θ that minimizes:


$$\underset{\theta }{\min}\;\sum F(x|\theta {)}^{2}-y^{2}.$$

Note that because we linearized performance in *d′* and normalized performance for each observer, this is mathematically equivalent to a classical generalized linear model with a probit linking function fitted through maximum likelihood [46]. To compute statistics on fit parameters, we resampled the data set 1,000,000 times and fitted the functions to each sample. *P*-values were doubled to reflect two-sidedness.

For cross-validation, we randomly selected 10 out of the 15 observers, resulting in 3,003 possible combinations of test and validation sets, and fitted each model to the mean performance in *d*′ once for each sample. We then computed the mean error of the remaining five observers as a validation score.

Psychometric functions for the Nonius task were fitted using maximum likelihood estimation. Finally, vergence was computed by subtracting the left and right eye position in visual angle relative to fixation. Student's *t*-tests on vergence eye postures were one sided and uncorrected for multiple comparisons. Vergence eye posture was smoothed by a 5-ms Gaussian kernel in Figs. 3D and S5.

## Supplementary Material

pgad314_Supplementary_Data

## Data Availability

Raw behavioral data for this study are openly available on the Open Science Framework website at DOI: 10.17605/OSF.IO/PMAHZ.
