# Supplementary material for: Orienting attention across binocular disparity
Source: PNAS Nexus. 2023 Sep 27;2(10):pgad314. doi: 10.1093/pnasnexus/pgad314 (PMC10563658; doi:10.1093/pnasnexus/pgad314)
Supplement: pgad314_Supplementary_Data [file pgad314_supplementary_data.docx]

**
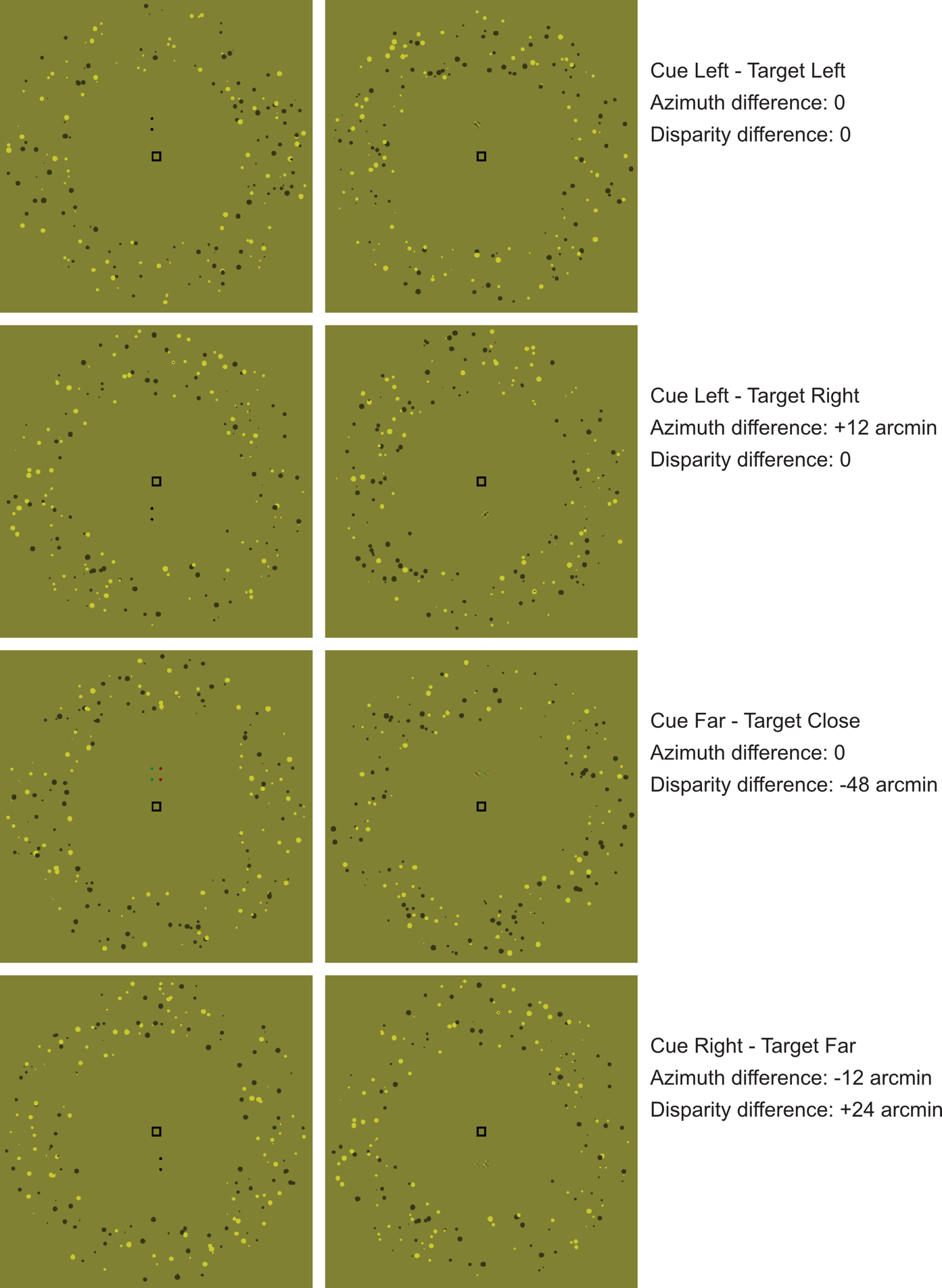
Figure S1:** Additional stimulus configuration examples. Each row depicts one example. Figures in the left column show attentional cuing conditions, and figures in the right column show the stereoscopic stimulus that followed the corresponding attentional cue. For each row, the text on the right describes the corresponding cue-stimulus pair.

**
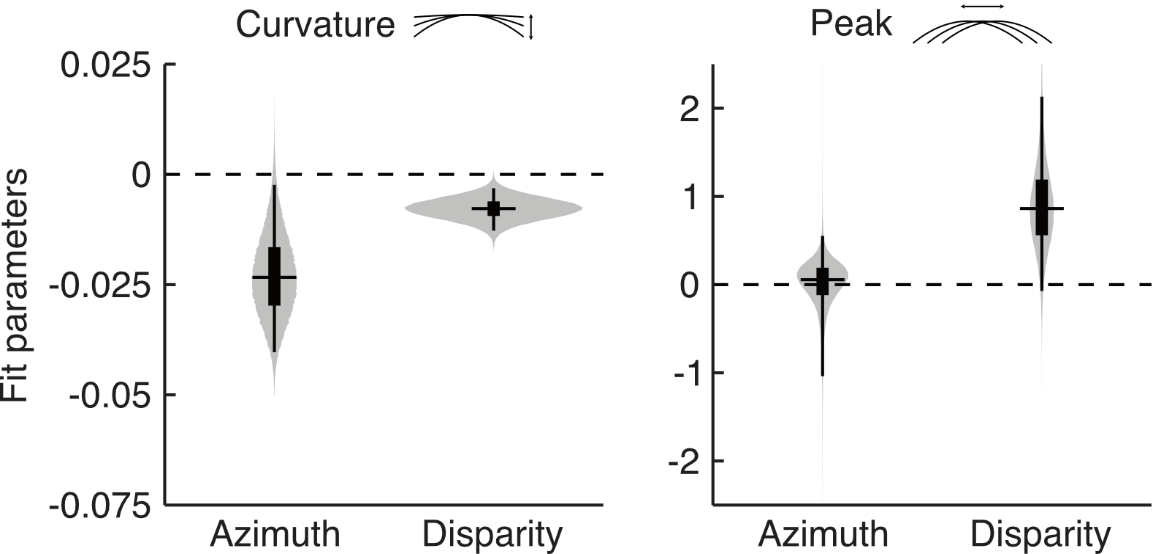
**

**Figure S2:** Distribution of fit parameters for the polynomial fits in Figures 2C and 2E. Horizontal lines are medians, thick vertical lines quartiles, thin vertical lines 95% confidence intervals, and shaded areas the full distribution of fit parameters from resampling. Performance as a function of azimuth or disparity was fitted with a polynomial *y=ax^2^+bx+c* (with x either azimuth or disparity). The term *a* corresponds to the curvature of the polynomial (left graph ordinate is *a*) and *-b/2a* corresponds to the peak location of the polynomial as a function of *x* (right graph ordinate is -*b/2a*).

**
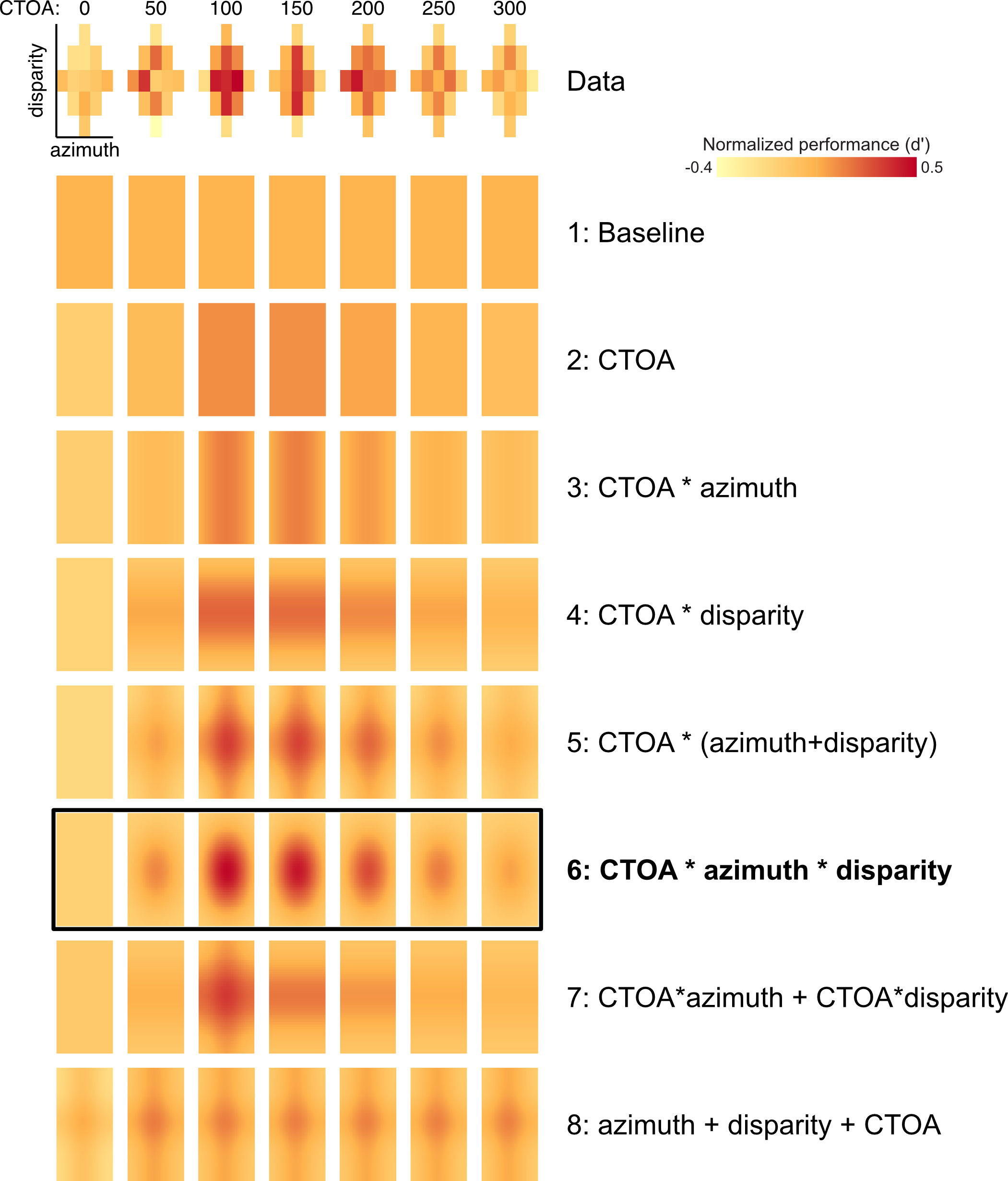
**

**Figure S3:** Depiction of the models used during model comparison. Top row: mean normalized performance (color) as a function of azimuth (abscissa), disparity (ordinates) and CTOA (panels). The following 8 rows show prediction from the different models tested by cross-validation. The black rectangle indicates the best model.


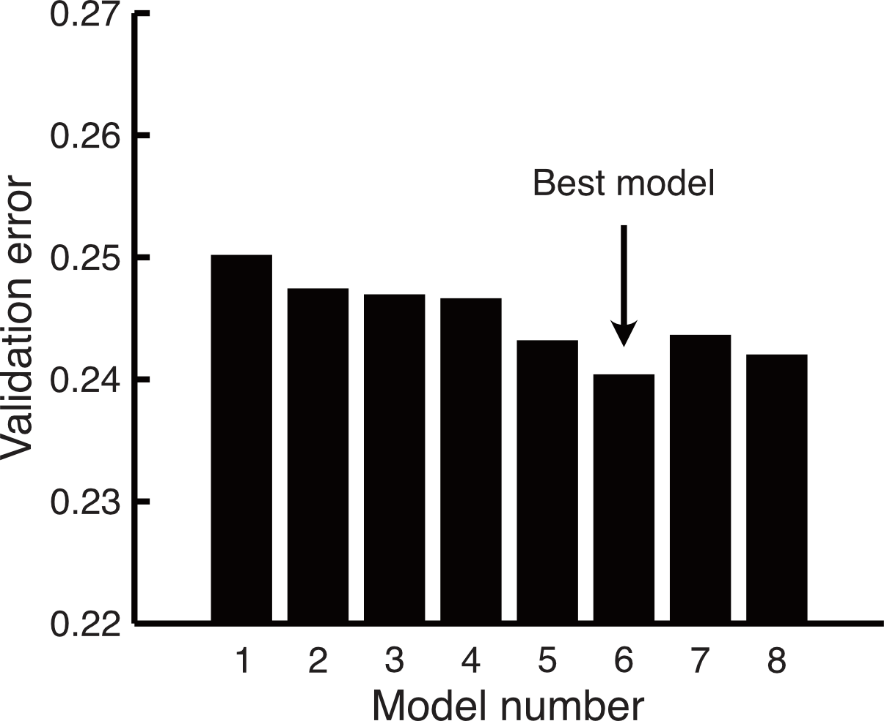


**Figure S4:** Validation error for the 8 models depicted in Figure S3. The model with the lowest validation error was model 6, assuming a multiplicative interaction between azimuth, disparity and time.


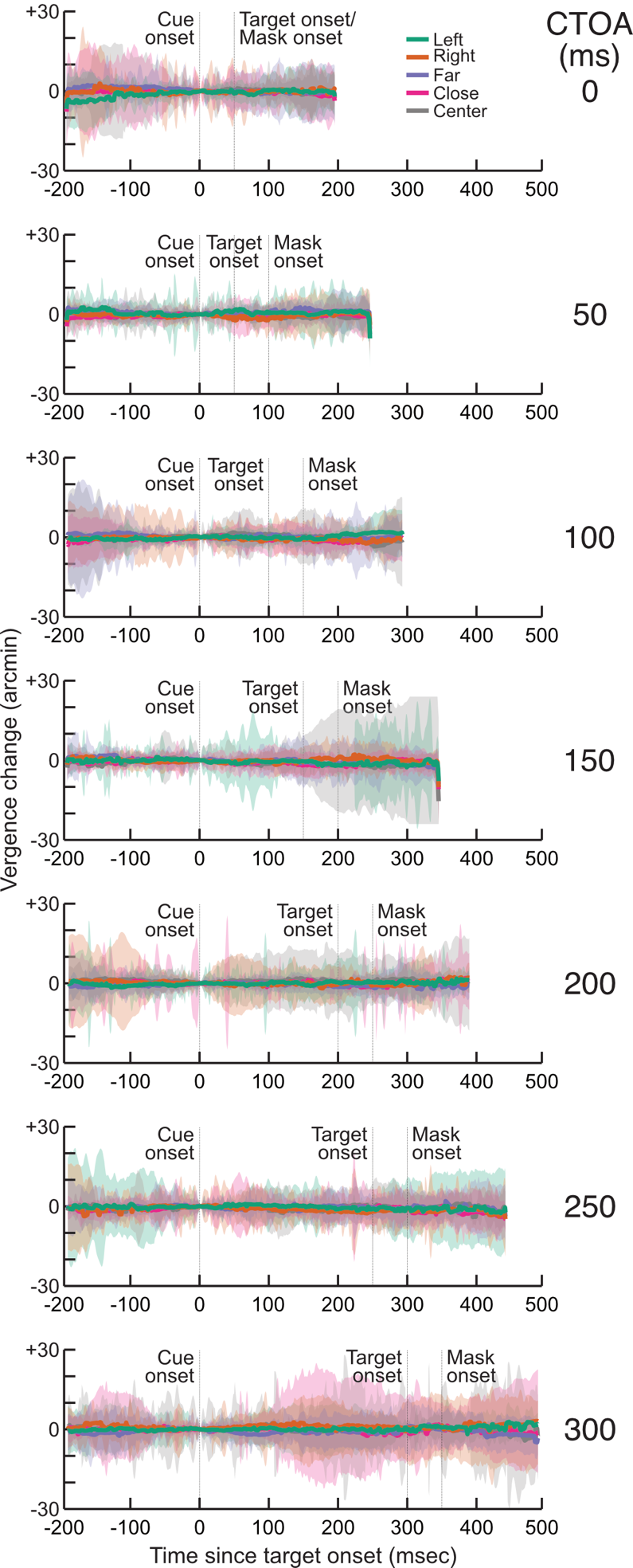


**Figure S5:** Average change in vergence eye-posture during attentional trials across observers (lines) relative to cue onset and standard error (shaded area) as a function of time (abscissa), cue location (colors) and CTOA (subplots).

**
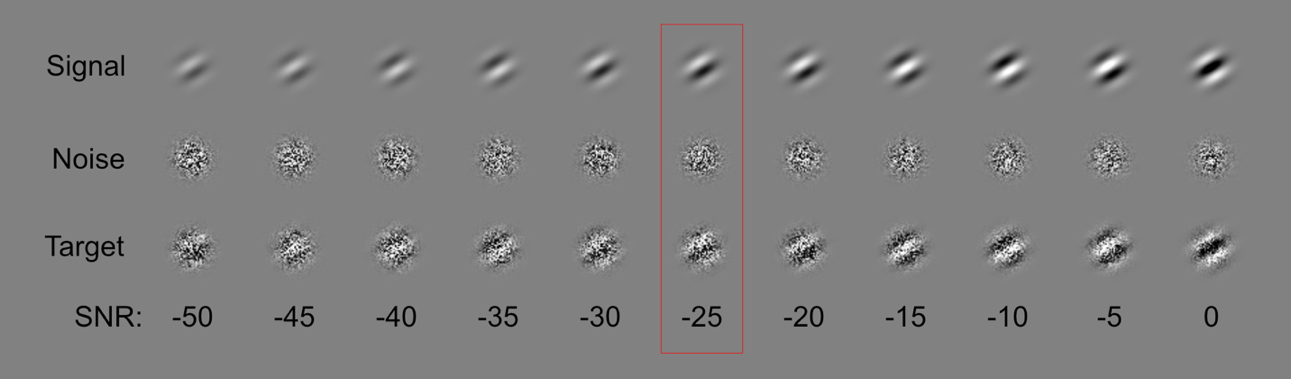
Figure S6:** Examples of noised Gabor-patch targets with varying levels of Signal-to-Noise Ratio (SNR). Noise contrast (RMS) is varied inversely to the signal intensity so as to maintain a fixed contrast for the Gabor patch. SNR is expressed using the standard definition of root-power decibels. The red frame indicates approximately the mean SNR across participants measured during the titration task.
